# A novel *MLH1* mutation in a Japanese family with Lynch syndrome associated with small bowel cancer

**DOI:** 10.1038/s41439-018-0013-y

**Published:** 2018-06-21

**Authors:** Yoshika Akizawa, Toshiyuki Yamamoto, Kazuo Tamura, Toshiyuki Kanno, Nobuko Takahashi, Takeshi Ohki, Teppei Omori, Katsutoshi Tokushige, Masakazu Yamamoto, Kayoko Saito

**Affiliations:** 1 Department of Obstetrics and Gynecology, Tokyo Women’s Medical University, Tokyo, Japan; 2 Institute of Medical Genetics, Tokyo Women’s Medical University, Tokyo, Japan; 3 Magor in Science, Graduate School of Science and Engineering Research, Kinki University, Higashiosaka, Japan; 4 Department of Surgery, Institute of Gastroenterology, Tokyo Women’s Medical University, Tokyo, Japan; 5 Institute of Gastroenterology, Department of Internal Medicine, Tokyo Women’s Medical University, Tokyo, Japan

## Abstract

Lynch syndrome is a genetic disorder related to cancer predisposition, including colorectal cancer, endometrial cancer, and ovarian cancer. Germline mutations in mismatch repair genes, including *MLH1*, *MSH2*, *MSH6*, and *PMS2*, are responsible for this condition. Cancer tissue specimens resected from small bowel adenocarcinoma in a Japanese patient showed decreased expression of MLH1 and PMS2 by immunohistochemistry testing. Finally, a novel *MLH1* mutation, c.1833dup, was identified in this patient.

Colorectal cancers (CRCs) are involved in major types of cancer in Japan, and more than 49,000 Japanese people with CRCs died in 2015^1^. Approximately 20~25% of patients with CRCs show a family history, and genetic factors account for 5~15% of all CRC patients^2^. Some genetic cancer syndromes are involved in this condition. In 1966, Lynch et al. reported two large families with hereditary nonpolyposis colorectal cancer (HNPCC; MIM #114500) to differentiate it from another inherited form of CRC^3, 4^. However, patients with HNPCC often present other cancers, suggesting a predisposition to multiple cancers. Thus, Lynch syndrome (LS; MIM #120435) is now a widely used alternative term.

LS is generally inherited as an autosomal dominant trait. Familial clustering of CRCs, an excess of synchronous or metachronous CRCs, and other extra colonic malignancies are often observed in the same family with LS. Germline mutations in mismatch repair (MMR) genes, including *MLH1*, *MSH2*, *MSH6*, and *PMS2*, are related to LS, with a penetrance of approximately 80% for CRCs, 60% for endometrial cancer, and less than 20% for other cancers^5^. Generally, molecular diagnosis will be offered to patients who fulfill the clinical diagnostic criteria as defined by the Amsterdam I/II criteria or revised Bethesda guidelines^4, 6^. If the patients receive a final diagnosis by molecular analysis, such information will be useful not only for themselves but also for their family members. Presymptomatic family members carrying the same variants are at risk of developing some cancers. Thus, they will be recommended for periodic health check-ups. However, if at-risk family members do not show the same variants, they will be released from the high-risk health check-ups.

Recently, we saw a new patient with LS who presented a novel mutation of *MLH1*. The proband (II-2) is a 43-year-old Japanese male. He was referred to the department of gastroenterology in our hospital for a medical check-up owing to microcytic anemia and persistent fecal occult bleeding. Endoscopy examination was performed, and small bowel cancer was suspected. Finally, a diagnosis of adenocarcinoma was obtained by endoscopic small bowel biopsy. Laparoscopic partial intestinal resection was performed under the diagnosis of jejunal cancer. Pathological examination confirmed moderately differentiated adenocarcinoma, and postoperative chemotherapy using capecitabine was administered. Later, recurrent cancer was identified in the mesenteric lymph node. Again, the small bowel was partially resected after lymph node dissection.

This patient has a remarkable family history. His father (I-1) died of rectal cancer at 41 years of age, and his elder sister (II-1) died of cancer of the corpus uteri and rectal cancer at 42 years of age (Fig. 1a). According to Amsterdam criteria II, we suspected LS as a potential candidate diagnosis. Three family members were affected, and all of them were first-degree relatives; they were diagnosed before 50 years of age, and two successive generations were affected. To confirm the clinical diagnosis, a molecular examination was performed.

**Fig. 1:**
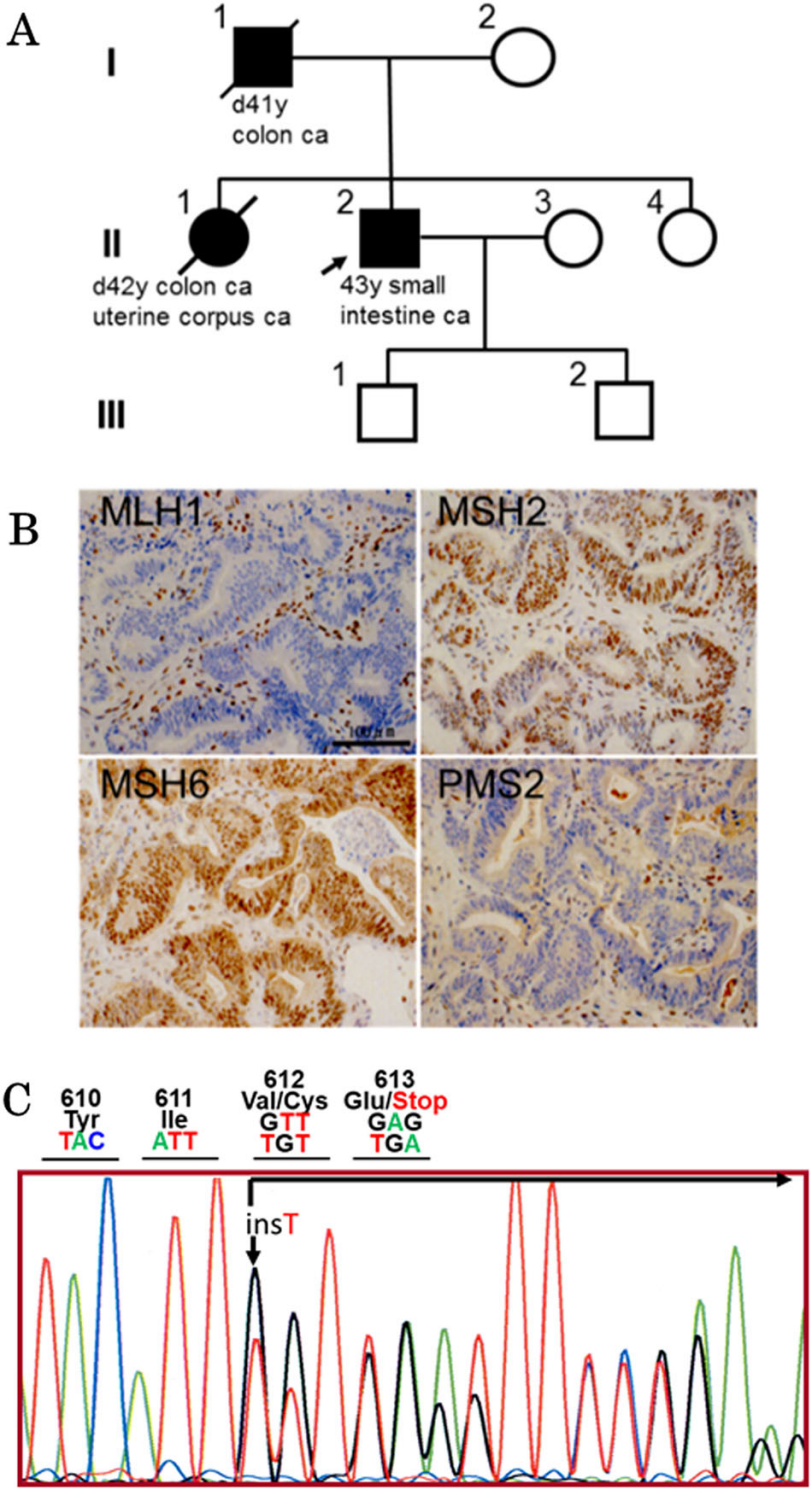
Clinical and laboratory information for the patient. ** a** Family tree of the patient (II-2). His father (I-1) and his elder sister (II-1) died from colon cancer and in association with uterine corpus cancer. **b** Immunohistochemistry testing for cancer tissue specimen showed negative staining for MLH1 and PMS2 but positive staining for MSH2 and MSH6. **c** Electropherogram of the Sanger sequence from the patient showing a single nucleotide (T) insertion in *MLH1*, c.1833-1834dup, changing codon 613 into a premature termination codon

This study was performed in accordance with the Declaration of Helsinki and was approved by the ethical committee of the Tokyo Women’s Medical University. After obtaining written informed consent, blood samples and tissue specimens from extracted small bowel cancer tissue were collected. First, immunohistochemistry testing was performed, and loss of MLH1 and PMS2 expression was determined (Fig. 1b). This suggested the involvement of the *MLH1* gene because MLH1 protein forms a dimer with PMS2 protein^7, 8^. For confirmation, Sanger sequencing analysis of *MLH1* was performed according to previously reported methods^9^. For this analysis, genomic DNA was extracted using a QIAamp DNA Mini Kit (Qiagen, Hilden, Germany). Finally, a possible pathogenic mutation, NM_000249.3 (MLH1_v001):c.1833dup [p.Val612Cysfs*2] (Fig. 1c), which leads to a premature termination codon, was identified (Fig. 1c). This mutation has not been registered in the InSiGHT variant database (https://www.insight-group.org/variants/databases/), in which 1344 *MLH1* variants are currently registered. ClinVar (https://www.ncbi.nlm.nih.gov/clinvar/) and the professional human gene mutation databases (HGMD) (http://www.hgmd.cf.ac.uk/ac/index.php) were also checked, and this variant was not included. Therefore, c.1833dup was considered a novel mutation.

Among four MMR genes, three genes (*MLH1*, *MSH2*, and *MSH6*) shared 96%, and *MLH1* mutations were the most prevalent^10^. The sensitivity of immunohistochemistry testing to detect MMR gene mutations has been reported to be 45%^11^. Therefore, identification of the *MLH1* mutation in this patient showing loss-of-function for MLH1 and PMS2 by immunohistochemistry testing is reasonable.

According to the database, most of the reported pathogenic variants in MMR genes lead to nonsense or premature termination^12^, which suggests that the pathogenic mechanism of MMR genes is mainly derived from a loss-of-function. Loss-of-heterozygosity likely occurs in cancer cells by second hit mutations in somatic cells. By this mechanism, the expression of MMR genes would be decreased, as shown by immunohistochemistry testing for cancer tissues.

In the present patient, the first disease manifestation was small bowel cancer, which is not rare in patients with LS^13, 14^. After careful genetic counseling and obtaining permission from the patient, his genetic information was disclosed to his partner and younger sister (II-3 and II-4).The younger sister (II-4) declined to be genotyped herself and was referred for periodic medical check-ups, which is acceptable because she has the right not to know her genotype^15, 16^. More time and more supports may be necessary for her for consideration^17, 18^. Regarding the offspring of the present patient under the age of adulthood, genetic counseling about their genetic testing to confirm their carrier status should be provided in the future.

In this study, we successfully identified a novel *MLH1* mutation in a Japanese patient with LS. This genetic information will be useful if the patient’s offspring are willing to be genotyped in the future.

## Data Availability

The relevant data from this Data Report are hosted at the Human Genome Variation Database at 10.6084/m9.figshare.hgv.2324.
